# Collaborative testing in physical examination skills training and the autonomous motivation of students: a qualitative study

**DOI:** 10.1186/s12909-021-02618-7

**Published:** 2021-04-21

**Authors:** Jiska A. Patiwael, Anje H. Douma, Natalia Bezakova, Rashmi A. Kusurkar, Hester E. M. Daelmans

**Affiliations:** 1 Amsterdam UMC, Vrije Universiteit Amsterdam, Faculty of Medicine, De Boelelaan 1117, Amsterdam, Netherlands; 2 Amsterdam UMC, University of Amsterdam, Faculty of Medicine, Meibergdreef 9, Amsterdam, Netherlands

**Keywords:** Collaborative testing, Clinical skills training, Motivation

## Abstract

**Background:**

Teaching methods that stimulate the active learning of students make a positive impact on several aspects of learning in higher education. Collaborative testing blended with teaching is one such method. At our medical school, a training session was designed using a collaborative testing format to engage medical students actively in the theoretical phase of a physical examination training, and this session was evaluated positively by our students. Therefore, we extended the use of the format and converted more of the training into collaborative testing sessions. The literature on collaborative testing and the theoretical framework underlying its motivational mechanisms is scarce; however, students have reported greater motivation. The aim of the current study was to investigate student perceptions of a collaborative testing format versus a traditional teaching format and their effects on student motivation.

**Methods:**

Year four medical students attended seven physical examination training sessions, of which three followed a collaborative testing format and four a traditional format.

The students were asked to evaluate both formats through questionnaires comprised of two items that were answered on a five-point Likert scale and five open-ended essay questions. Content analysis was conducted on the qualitative data. The themes from this analysis were finalized through the consensus of the full research team.

**Results:**

The quantitative data showed that 59 students (55%) preferred collaborative testing (agreed or strongly agreed), 40 students (37%) were neutral, and 8 students (8%) did not prefer collaborative testing (disagreed or strongly disagreed).

The themes found for the collaborative testing format were: ‘interaction’, ‘thinking for themselves’, and ‘active participation’*.* ‘Interaction’ and ‘thinking for themselves’ were mainly evaluated positively by the students.

The most frequently mentioned theme for the traditional format was: ‘the teacher explaining’. Students evaluated this theme both positively and negatively.

**Conclusions:**

The most frequently mentioned themes for the collaborative testing format, namely ‘interaction’, ‘thinking for themselves’, and ‘active participation’, fit within the framework of self-determination theory (SDT). Therefore, the collaborative testing format may support the fulfilment of the three basic psychological needs indicated in SDT: autonomy, competence, and relatedness. Thus, our findings provide initial support for the idea that the use of collaborative testing in medical education can foster the autonomous motivation of students.

## Background

### Collaborative testing

Teaching methods that stimulate active learning have been shown to make a positive impact on the self-regulated motivation, engagement, retention, and achievement of students in higher education [1–3]. Retrieval practice by means of testing is one such a method, and it is one of the most effective ways for learning, knowledge retention, and application of information [4]. Collaborative testing is defined as ‘a student-focused, active learning strategy as well as an interpersonal form of critical thinking’ [5]. In a survey study, students reported that, they used this learning method when studying in a group environment [6]. However, studies on collaborative testing are few, and although students have reported greater motivation following collaborative testing [7, 8], the literature on the theoretical framework underlying its motivational mechanisms is scarce. Therefore, the aim of this study was to investigate student perceptions of both collaborative testing and a more traditional teacher-centred format and their preferences.

Interest in collaborative testing has increased in recent years for both medical and nursing education. Collaborative testing blends collaborative learning and assessment. Students work together in small groups and explain their reasoning, share knowledge, and integrate different perspectives, thus also practising interpersonal skills [9]. These skills are important for medical students to develop for use in future clinical practice [10]. Collaborative testing is described as relying on constructivist learning theory [5] in order to engage students in their own learning. Constructivist learning theory posits that learners actively construct knowledge and make meaning based on their experiences, individually or socially [11, 12]. Collaborative testing can be a one-stage (single group exam) or two-stage process (individual exam followed by group exam) [5].

### Self-determination theory (SDT)

SDT is a theory of motivation developed by Ryan and Deci. In SDT, students are viewed as active organisms acting on their environment rather than as passive recipients [13]. Three basic psychological needs are central to this theory: the experiences of autonomy (engaging in an activity of choice), competence (feeling capable and effective in producing desired results and utilizing one’s capacities), and relatedness (feeling connected to others or belonging to a social environment).

Furthermore, SDT distinguishes between different types of motivation. These vary on a qualitative scale from a lack of motivation (called ‘amotivation’) through different forms of extrinsic motivation to intrinsic motivation (IM). Extrinsic motivation consists of external regulation (regulation through punishments and rewards), introjected regulation (living up to expectations, feelings of shame, feelings of guilt), identified regulation (realizing and believing in the importance of a rule), and integrated regulation (internalizing rules along with one’s own norms and values). IM is a form of motivation that creates the free involvement in an activity out of personal interest. External and introjected forms of regulation are often called ‘controlled self-regulation’, while identified, integrated, and fully intrinsic forms of regulation are referred to as ‘autonomous self-regulation’ [14, 15].

Satisfaction of the three basic psychological needs stimulates autonomous motivation, meaning that the ‘learner learns out of genuine interest or personal value’ [16, 17].

Autonomous motivation in medical education has been associated with better learning outcomes and less exhaustion than controlled motivation [18, 19]. Learning in small groups where students work collaboratively on problems has been cited as an example of a teaching-learning method that increases self-determined motivation in students [15].

### Clinical skills training

To our knowledge, the clinical skills training conducted in skills labs has not yet been investigated in relation to active learning principles. Duvivier et al. suggested that a more active and student-centred approach to clinical skills teaching might be more suitable than a more traditional approach such as teacher-centred learning [20].

We designed a collaborative testing format in order to engage students actively in the theoretical phase of one of their physical examination training sessions. In a pilot study in 2017 (*n* = 100), we investigated how medical students would value this new teaching format through a qualitative study. The students evaluated the collaborative testing format positively. They were mainly positive about the opportunities for interaction and independent thinking. We, therefore, extended the collaborative testing format and converted more of the physical examination training into sessions using collaborative testing.

The research questions of the present study were:

What perceptions do students have of collaborative testing and traditional teaching formats in the theoretical phase of physical examination training? What are their preferences and why?

## Methods

### Study context

At the start of the master’s program and before entering clinical clerkships, medical students at VU University attend a six-week transition course at a clinical skills centre. During the first 3 weeks of this transition course, students are trained in consultation skills and physical examination.

From June to September 2018, the students included in the present study attended three physical examination training sessions that used a collaborative testing format and four using a traditional teaching format.

In the collaborative testing sessions, the training focused on the physical examination of the head and neck, abdomen, and peripheral vascular system. In the traditional training sessions, the training focused on the general examination, lungs, heart, and breasts. There were 12 students in each group.

### Participants

In total, 114 students participated in the physical examination training sessions.

### Preparatory reading

Prior to each training, the students were advised to prepare by reading the relevant chapter in the textbook. Pre-training reading was voluntary.

### Structure of the collaborative testing format

**Fig. 1:**
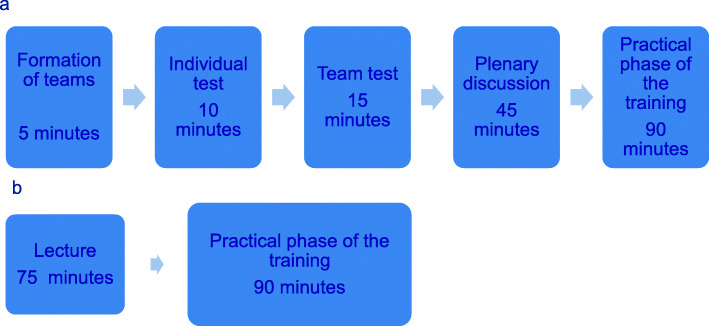
Different phases of the collaborative testing (1**a**) and traditional (1**b**) teaching formats

The collaborative testing format had five phases: the formation of teams, individual test, team test, plenary discussion, and practical phase of the training (Fig. 1a). The total duration of the training was 3 h. All the facilitators were clinical skills teachers as well as medical doctors.

#### The formation of teams

At the start of the training, the students were asked to form into three teams of their choice consisting of four students per team (5 min).

#### Individual test (iT)

During the first 10 min, the students’ individual prior knowledge (from the preparatory reading) was tested using multiple choice questions (MCQ) with a single best answer format.

#### Team test (tT)

For the next 15 min, the same MCQ test was completed by the preformed teams immediately following the iT with the intent of promoting discussion and allowing the students to see more than their own individual perspectives. Each team chose a team captain to guide the discussion. The goal of the discussion was to reach a consensus on the answers to all of the questions. The use of laptops or books was not allowed during the iT or tT.

#### Plenary discussion

For the next 45 min, the facilitator asked each of the team captains to provide the answers to all of the questions. After registering the answers, the team captain or another team member explained the team’s reasoning. The facilitator then gave the correct answer, registered the team scores, and clarified incorrect answers, thereby giving feedback to the students immediately. The team with the highest score was then declared. During this phase, the students were allowed to look up the relevant medical topics using laptops or books if necessary.

#### Practical phase of the training

In the final phase of the physical examination training (90 min), the teacher demonstrated the examination. Afterwards, the students practiced the physical examination skills on each other under supervision.

### Structure of the traditional format

The traditional format contained two phases: a lecture and the practical phase of the training (Fig. 1b). The total duration of the training was 3 h, and the training was provided by clinical skills teachers who were all medical doctors.

#### Lecture

In the first phase of the physical examination training, the clinical skills teacher provided the students with the background knowledge on how to perform the skills and on how to interpret normal and pathological findings (75 min).

#### Practical phase of the training

The second phase of the physical examination training (90 min) was identical to that of the collaborative testing format.

### Data collection and analysis

#### Student survey

Two paper-based questionnaires, one for the traditional format and the other for the collaborative testing format, were distributed to the student participants 3 days after the completion of the seven physical examination training sessions (at the start of week three). The questionnaires included five open-ended essay format questions and the following two statements for rating: ‘I preferred the new teaching format over the traditional teaching format’ and ‘I preferred the traditional teaching format over the new teaching format’ (five-point Likert scale: strongly disagree, disagree, neutral, agree, strongly agree) (Table 1) [21, 22]. The questionnaires underwent a peer review (by a faculty member at the institute) and expert review (two educational experts). The qualitative data were collected until data sufficiency was reached, and then a content analysis was conducted [23]. The qualitative data from the descriptive answers were open coded and a consensus was reached on the themes through iterative discussions among the four members of the research team. Once the data were coded and categorized, the data within each theme were quantified in order to measure their thematic prevalence. Based on the words that the students used, the data were labelled as positive, negative, or neutral. When students used both positive and negative words, the data were labelled as both positive and negative. The ratings on the statements were collected, frequencies including percentages were calculated, and a frequency distribution was made.

**Table 1:** Open-ended essay format questions and rating statement for the collaborative testing format^a^

We would like to ask you to elaborate on the following items based on your experience of the physical examination training sessions using the new teaching format. Please give your opinion on:	
Your role during the training sessions.	
The role of your colleagues during the training sessions.	
The role of the clinical skills teacher during the training sessions.	
What was the most positive learning aspect of the new teaching format?	
What was the most negative learning aspect of the new teaching format?	
I preferred the new teaching format over the traditional teaching format (5-point Likert scale: strongly disagree, disagree, neutral, agree, strongly agree).	

^a^For the traditional teaching format, the same questionnaire was used

Ethical approval: The ethical review board of the Netherlands Association for Medical Education (NVMO) was asked for an ethical review of the research proposal. The board concluded that, since all the data were to be collected in the course of regular program evaluations and were anonymous, no further ethical review was necessary, and they approved the conduction of the study (NVMO-ERB, file no. 2018.6.1).

Informed consent was obtained from all participants, and the participants were informed that participation was voluntary and that they could withdraw from the study at any time. No incentives were provided. Data were collected anonymously in the course of the regular program evaluation, and the evaluation method was in accordance with the guidelines and regulations within the Faculty of Medicine, VU University, Amsterdam University Medical Centre.

## Results

A total of 113 (99% response rate) students filled out the questionnaires. The reason for the non-participation of the one missing student is not known. Only 95% (107/113) of the forms were available for the analysis as six that were filled out incorrectly had to be excluded.

We were able to identify the following themes for the collaborative testing (Table 2), and based on the responses to the questions, the answers were labelled as positive and/or negative, or neutral. When labelled both positive and negative, it was marked in both columns.

**Table 2:** Frequencies of the themes from the qualitative data for both teaching formats

Collaborative testing format				Traditional teaching format			
Theme	Negative	Neutral	Positive	Theme	Negative	Neutral	Positive
Interaction	1	26	61	The teacher explaining	2 79 (Both + and -)	4	20 79 (Both + and -)
Thinking for themselves	1	7	47	Listening	6 1(Both + and -)	61	2 1(Both + and -)
Active participation	1	28	33	Questions	1 1 (Both + and -)	55	7 1(Both + and -)
				Structure			29
				Passive/ less active	21 4 (both + and -)	34	2 4 (both + and -)

### Interaction

The students mainly evaluated this theme positively. They described how they discussed a topic or thought along with their peers and learned from each other: ‘*and then discussing with fellow students, that makes you think at a deeper level*’*.* They also described how they valued working together in teams: ‘*to work together and to formulate an answer*’*.*

### Thinking for themselves

Students mainly evaluated this theme positively. They described how they thought about each topic alone first:

‘*It really gets you thinking, and it is a good thing that you do not search for the answer right away but really think in a logical manner first*’*.*

### Active participation

Students mentioned that they were active in the collaborative testing: ‘*active mentality, therefore, you participate more*’*.* Active participation was evaluated both positively and neutrally.

We identified the following themes for the traditional format (Table 2):

### The teacher explaining

The students evaluated this theme both positively and negatively. Students said that they were provided a great deal of useful information and a clear explanation by the teachers: ‘*The teachers take their time to explain something well and to lead you through the subject matter*’*.*

On the other hand, the students described that they did not think about the topic for themselves first: ‘*You have to listen a lot, that’s why you think less for yourself*’. The students also commented on the fact that they were more passive: ‘*Because of this…. (much was being explained, as in a lecture) we were handling the content material less actively…*’ and that there was less interaction: ‘*The interaction is lacking*’*.*

Furthermore, some students described that, after a while, their concentration lessened, and other students described the lecture as tedious when the teacher was explaining: ‘*After a while, the concentration diminishes because of the fact that a lot of explaining has been done and the student’s participation is not elicited*’*,* ‘*...hard to listen passively for that long, I had to keep myself awake’,* and *‘it can be rather boring or dull*’*.*

### Listening

The students often mentioned this theme when commenting on the traditional format. They mostly evaluated this theme neutrally: ‘*a student listening*’. Some students evaluated this theme negatively: ‘*only listening and registering*’*.*

### Questions

‘The student asking the teacher questions or vice versa’ was mentioned as a neutral part of the traditional format: ‘*asking questions*’. Some students evaluated this theme positively: ‘*there is always room for questions’*.

### Structure

Structure was mentioned as part of the traditional format. Students evaluated this theme positively: ‘*clear structure in which we were offered the information. Good to have it organized*’*.*

### Passive/less active

A passive/less active mentality was mentioned as part of the traditional format. Students were mainly neutral or negative about this theme. *‘less active’* and *‘less input by the student, a more passive student’.*

### Preference

**Fig. 2:**
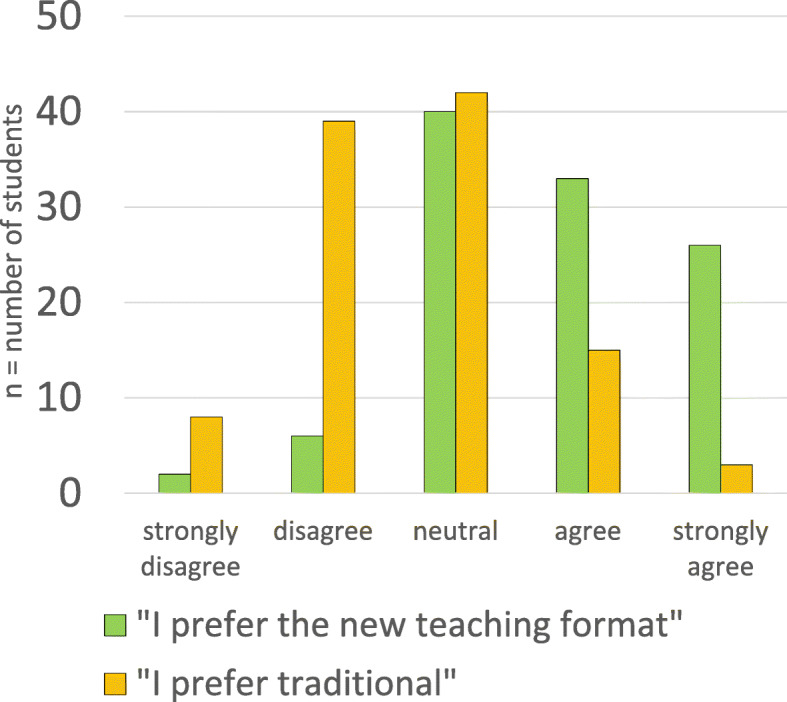
Likert scale survey on student preferences

A total of 59 of the students (55%) reported a preference (agree or strongly agree) for the collaborative testing format. See Fig. 2 for the findings of the survey on student preferences.

## Discussion

**Fig. 3:**
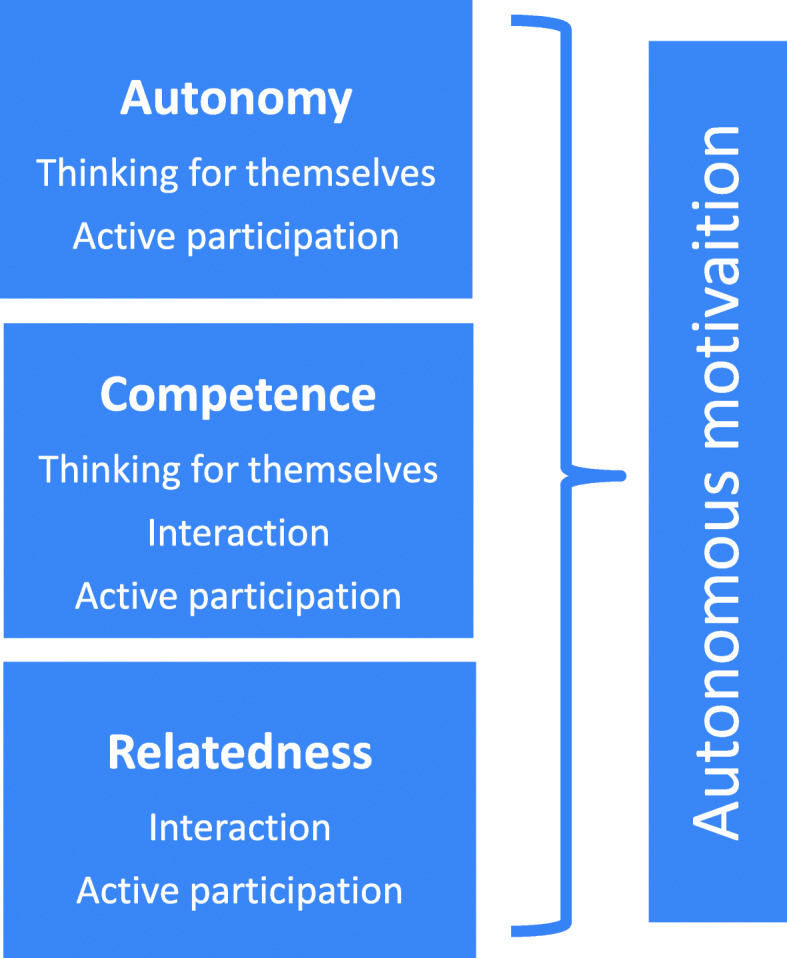
Diagram of the three basic psychological needs of autonomy, competence, and relatedness. The three basic psychological needs are supported within the three collaborative testing format themes of ‘thinking for themselves’, ‘interaction’, and ‘active participation’, thus enhancing autonomous motivation

The lens of SDT provides a useful framework for understanding student perspectives [14, 24]. Concerning the collaborative testing format, three major themes emerged: thinking for themselves, interaction, and active participation. Within these themes, the three basic psychological needs of autonomy, competence, and relatedness were supported for a number of the students, thus enhancing autonomous motivation (see Fig. 3).

### Thinking for themselves: autonomy and competence

Autonomy: Many students mentioned that ‘thinking for themselves’ was the most positive learning aspect of the collaborative testing format. This may indicate that these students participated *voluntarily* and that they *wanted* to think about the questions in the iT *without external or internal pressure* to carry out the activity. Engaging in an activity of choice creates an experience of autonomy, one of the three basic psychological needs [14].

Furthermore, it most likely also indicates that they participated in the activity because they were aware of its importance. In a qualitative study on the strength of collaborative testing, students reported being more motivated to prepare out of a sense of responsibility [7]. This is indicative of autonomous motivation [25].

In our study, we provided a meaningful rationale when advising the students to prepare for the training in advance and when asking them to take the iT, as Reeve suggested [26]. However, even if the students did not prepare, they could still participate in the training. It was further mentioned to the students beforehand that the iT was for their own use and no grades were to be given on it, which is contrary to how collaborative testing is mostly used [27]. In this way, we prevented the creation of a feeling of external or internal pressure to perform an activity. Soenens and Vansteenkiste have emphasized that it is important to avoid imperative language to ensure autonomy support in teaching [28]. During their bachelor’s program, the students gained some knowledge and skills on how to perform physical examinations and interpret normal and pathological findings. Therefore, they were able to think and reason about the questions, even if they were not specifically prepared for the training. Moreover, activating prior knowledge is essential for anchoring new subject material [29].

A clear structure was provided in the collaborative testing format (see Fig. 1). Jang et al. reported that in order to engage students in learning activities, autonomy support as well as structure is necessary [30]. Furthermore, Sierens et al. described that autonomy with structure is important for the self-regulation of learning [31].

The students mentioned that while they were ‘thinking for themselves’ in the collaborative testing sessions, they were challenged to think about the subject matter. Similarly, Kusurkar and Croiset have reported that autonomous motivation is associated with optimal challenge [25]. Furthermore, the students described that in the traditional teaching format, they put less effort into thinking for themselves. Along the same lines, Chang et al. argued that passive-learning environments have fewer possibilities for offering optimal challenges [32].

Competence (the second basic psychological need): The collaborative testing format consisted of three different steps. First the individual test, then the team test, and finally the plenary discussion. Some students spontaneously and explicitly mentioned that first thinking for themselves and subsequently discussing the questions with their peers was a positive learning aspect of the collaborative testing format. When students think for themselves, they can experience how competent they are in the subject matter. Likewise, Kusurkar suggested that teachers can facilitate a gradual building of competence in students by breaking down tasks into smaller steps [24]. Situations offering problem solving situations that are within the reach of the abilities of students can make them feel autonomous and competent [33, 34]. Furthermore, when a teaching method provides support for the students’ basic psychological needs of autonomy and competence, autonomous motivation is facilitated [17].

### Interaction: relatedness and competence

Relatedness: Interaction was a major theme in the collaborative testing sessions. The students described that they valued learning to work together in teams. In the traditional sessions, the students indicated missing a form of interaction. Both sessions, collaborative testing and traditional, involved small group training. In addition, the students had other small group training sessions with the same group of 12 students during the six-week transition course. These training sessions, in which the students got to know each other personally and the teacher got to know the students, were able to give the students a feeling that they belonged in a given social environment, gaining feelings of relatedness, the third basic psychological need. The students working together in teams in the collaborative testing session may have created an extra connection to the other students in the group. In a study on collaborative testing among medical students that used a mixed-methods convergence design, a number of students described that they had been engaged in processes that promoted teamwork [9]. In another study, which used a quasi-experimental design with a comparison group to examine the effects of collaborative testing as a learning strategy, nursing students reported positive interactions and collaboration with their peers [35].

Competence: The students in our study described that by discussing the questions in teams, they learned from each other. Therefore, these discussions increased the students’ competence. A study by Eastwood et al. also found that discussion during collaborative testing promoted learning from peers [9]. When the students in our study explained the content to their peers, their feelings of competence may have been strengthened by a perception of mastery over the content [14]. In a survey on the use of collaborative testing by Duane et al., faculty members observed that nursing students developed a knowledge base by discovering why some answers were accurate and why other answers were not [5]. By learning how the other students analysed the questions, they also improved their own critical thinking skills. Furthermore, the students in our study were given feedback by the facilitator during the plenary discussion and some students explicitly elaborated in the questionnaire on the fact the facilitator did so. When giving feedback, the facilitators in our study focused on doing so in a non-threatening way [36]. Hattie and Timperley recommended providing constructive feedback in a timely manner during a student’s learning process to demonstrate the knowledge gap [37].

### Active participation: autonomy, competence, and relatedness

A majority of the students mentioned that they were more active during the collaborative testing sessions. It is a generally accepted view that active learning allows students to engage in activities and think for themselves, increasing student performance [1]. Although the students appreciated that useful information was provided by the teacher in the traditional sessions, they also described that they were less active and after a while, their concentration diminished. Previous research has shown that the attention of students when listening to a lecture tends to become distracted after 15–20 min [38].

One of the 12 tips for engaging teachers in autonomy supportive teaching behaviours is to encourage active participation from students [36]. In addition, active learning involves activity and engagement while conducting meaningful learning tasks [39]. If active in-class participation from students is encouraged, it makes learning more autonomous and providing feedback easier while also increasing feelings of relatedness amongst the students and teacher [36]. Thus, active learning may have a positive effect on all of the three basic psychological needs, that is, autonomy, competence, and relatedness.

#### Preference

The students in our study preferred the collaborative testing format. Some students (*n* = 12) explicitly and spontaneously mentioned that they preferred a variation in teaching formats, for example the use of both traditional teaching and collaborative testing.

### Limitations

In this study, the students were not placed in the same teams during the three collaborative testing sessions. Allocating students to the same team for all collaborative testing sessions would have helped in strengthening the feeling of relatedness to the group. On the other hand, letting students choose their own teams may have supported feelings of autonomy. Our physical examination trainings were conducted in a time span of 3 weeks, and this is a relatively short period of time. If the students had worked together for a longer period of time, they might have also strengthened their feelings of relatedness to the group. However, we did find interaction as a theme in our qualitative data; therefore, relatedness seemed to be stimulated by the collaborative testing sessions even though the trainings were planned in a relatively short period of time. Our study was performed as part of the regular master’s program and therefore has a higher ecological validity than studies performed in an artificial setting. Ecological validity refers to the ability to generalize study findings to real-world settings. However, despite the high ecological validity, results still cannot be generalised to other university educational settings.

### Future implications

Future research should be comprised of both a focus group study to explore student views in greater depth and a quantitative study to further investigate the extent of the motivational implications of collaborative testing in physical examination training.

## Conclusions

The students preferred the collaborative testing format over the traditional format. We found that the three basic psychological needs of autonomy, competence, and relatedness were supported by the physical examination collaborative testing format. Thus, this study provides support for the motivational implications of collaborative testing in a medical education context.

## Data Availability

The datasets used and analysed during the current study are available from the corresponding author on reasonable request.

## Abbreviations

IM: Intrinsic motivation
iT: Individual test
MCQ: Multiple choice questions
NVMO: Netherlands association for medical education
SDT: Self-determination theory
tT: Team test
